# Individual nutrients and serum klotho levels in adults aged 40–79 years

**DOI:** 10.1002/fsn3.3310

**Published:** 2023-03-08

**Authors:** Sergej M. Ostojic, Elisabet R. Hillesund, Nina C. Øverby, Frøydis N. Vik, Anine C. Medin

**Affiliations:** ^1^ Department of Nutrition and Public Health University of Agder Kristiansand Norway

**Keywords:** aging, alcohol, carbohydrates, dietary exposure, klotho, NHANES

## Abstract

Several dietary factors (including adherence to the Mediterranean diet or higher nut intake) seem to positively affect circulating antiaging Klotho protein levels; yet, a description of possible relationships between individual nutrients and Klotho activity has not been evaluated. We analyzed the association of dietary intake of individual macro‐ and micronutrients and nonnutritive food components with circulating Klotho levels in a sample of 40‐ to 79‐year‐old US adults. Data from the 2015–2016 National Health and Nutrition Examination Survey were analyzed. Nutrient/food component intakes were calculated in relation to total energy intake using the nutrient density method, and available pristine serum samples were analyzed for serum Klotho concentrations. The final study sample consisted of 2637 participants (mean age 59.0 ± 10.7 years; 52% women). Higher Klotho concentrations were found with higher intake of carbohydrates (*p* < .001), total sugars (*p* < .001), dietary fibers (*p* < .001), vitamin D (*p* = .05), total folates (*p* = .015), and copper (*p* = .018). The results of the regression analysis with a crude model showed significant associations among five nutrients/food components (carbohydrates, alcohol, total sugars, dietary fibers, and niacin) and soluble Klotho levels across the sample. After adjusting the models for age and gender, the nutrient/food component–Klotho association remained significant for carbohydrates, total sugars, and alcohol (*p* < .05). Dietary exposure to individual nutrients and nonnutritive food components appears to be associated with Klotho activity; however, additional research is needed to investigate the relationship between cause and effect in diet composition–Klotho interplay.

## Graphical abstract

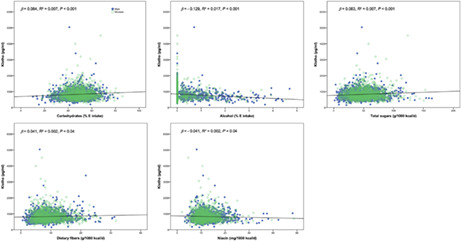

## 1 INTRODUCTION

Klotho (HFTC3) is a pleiotropic protein that plays many roles in human physiology, from modulating molecular aging via the activation of fibroblast growth factors‐related pathways (Kuro‐O, 2019), to controlling cellular uptake and homeostasis of various compounds, including calcium, phosphorus, and glucose (Torres et al., 2007). Klotho is often seen as an aging suppressor and a metabolic protector against several pathogenic processes (Dërmaku‐Sopjani et al., 2013). The prevention of Klotho decline can, therefore, be a novel therapeutic strategy for many age‐related diseases (Kuro‐O, 2019). Klotho exists in two forms, as a membrane‐bound enzyme/transporter and as a soluble hormone‐like carrier, with the circulating form often used as a proxy for the turnover of Klotho proteins (Olauson et al., 2017) and a biomarker of aging (Veronesi et al., 2021). Klotho expression appears to be negatively affected by age, bone loss, and alcohol consumption (Chalhoub et al., 2016; González‐Reimers et al., 2018). Several dietary factors (including adherence to the Mediterranean diet and/or nut intake) seem to positively affect Klotho levels (Jurado‐Fasoli et al., 2021; Jurado‐Fasoli, Amaro‐Gahete, De‐la‐O, Martinez‐Tellez, et al., 2019). Klotho activated by nutrition or other factors might further affect several metabolic processes, including bioenergetics balance (Ostojic, 2021). Previous studies linking Klotho with diet have originated from animal research or recruited a small number of human participants, without a description of possible relationships between specific nutrients and Klotho activity. Therefore, the main aim of the present cross‐sectional study was to analyze the association of dietary intake of individual macro‐ and micronutrients in addition to nonnutritive food components with circulating Klotho levels in a large sample of US adults using data from the 2015–2016 National Health and Nutrition Examination Survey.

## 2 METHODS

### 2.1 Study participants

The National Health and Nutrition Examination Survey (NHANES) is a regular biannual research program conducted by the National Center for Health Statistics (NCHS), a part of the Centers for Disease Control and Prevention of the U.S. Department of Health and Human Services (DHHS). NHANES was first designed in 1960 to assess and monitor the health and nutritional status of adults and children in the United States, with rounds continuously conducted from 1999 onwards in 2‐year cycles. NHANES combines in‐home personal interviews, physical examinations, and laboratory tests in mobile examination centers, with the survey targeting US civilian noninstitutionalized population aged 0–80 years. The sample for NHANES is selected using a complex four‐stage sample design, in which sample weights were used to produce estimates of health‐related statistics that would have been obtained if the entire sampling frame had been surveyed (Chen et al., 2020). In the 2015–2016 round, 15,327 persons were selected for NHANES from 30 different survey locations. Of the selected, 9971 completed the interview and 9544 were examined. For this article, we selected data from respondents who provided information about dietary intake and were examined for serum Klotho levels. The ethical approval to conduct the current round of NHANES 2015–2016 was granted by the NCHS Research Ethics Review Board (Continuation of Protocol #2011–17) and informed consent was obtained from all respondents.

### 2.2 Dietary assessment

Detailed dietary intake data from the NHANES 2015–2016 cohort were obtained through a dietary interview component. All NHANES participants were eligible for two 24‐hour dietary recall interviews. The first dietary recall interview was collected in‐person in the mobile examination center, and the second interview was collected by telephone 3–10 days later. For this article, we used the mean energy and nutrient/food component intakes of the two dietary recalls for each individual, from their Total Dietary Intake Data registry including foods and beverages as well as water. The calculated intakes did not include nutrients obtained from dietary supplements or pharmacological agents. Nutrient/food component intakes are presented in absolute numbers, and also in relation to total energy intake using the nutrient density method; for major macronutrients, nutrient densities are expressed as the proportion of the total energy intake, and for micronutrients and other food components, nutrient density was expressed as intake in appropriate units per 1000 kcal. The dietary interview component was conducted as a partnership between the U.S. Department of Agriculture (USDA) and DHHS. In this partnership, NCHS, Division of Health and Nutrition Examination Surveys, was responsible for the survey sample design and all aspects of data collection, and USDA's Food Surveys Research Group was responsible for dietary data collection methodology, maintenance of the databases used to code and process the data, and data review and processing (CDC/National Center for Health Statistics, 2018).

### 2.3 Serum klotho analyses

Only participants from the NHANES 2015–2016 cohort who provided dietary intake data and were examined for soluble Klotho levels were included in the final sample. Available pristine serum samples from 40‐ to 79‐year‐old participants in NHANES 2015–2016 cycles were analyzed for soluble Klotho levels with the IBL ELISA method (assay sensitivity 6 pg/mL). The Northwest Lipid Metabolism and Diabetes Research Laboratories, Division of Metabolism, Endocrinology, and Nutrition, University of Washington, performed the Klotho analyses. The final data set was additionally reviewed for completeness, consistency, and illogical values. Further details of the NHANES 2015–2016 round data protocol and procedures are documented elsewhere (CDC/National Center for Health Statistics, n.d.).

### 2.4 Demographics

NHANES 2015–2016 Demographics Data and Body Measures components were explored to acquire data on the general characteristics of participants, including individual, family, and household‐level information, and weight, height, and body mass index data.

### 2.5 Statistical analyses

Descriptive statistics were used to explore the distribution of Klotho levels in the sample, sample characteristics, and dietary intake. One‐way ANOVA was used to compare serum Klotho values across quartiles of dietary intake for each nutrient/food component (except for alcohol where we compare data below or above the median), with post hoc pairwise comparison tests employed to identify differences between individual sample pairs. The Kruskal–Wallis H test was used for testing trends across quartile categories. Bivariate analyses were used to identify relevant covariates, with the regression models adjusted for a posteriori recognized set of covariates. To avoid confounding of the nutrient–Klotho association due to variation in absolute food intake related to physical activity level and/or body size, we applied nutrient intake relative to energy intake as an independent variable in all models. Simple (crude) and multivariate linear regression analyses were performed to test the association between energy‐adjusted individual nutrients/food components and serum Klotho levels. An interaction term (gender x dietary constituent) was included in each model in addition to gender and the specific dietary component, to identify potential interactions by gender on the nutrient/food component–Klotho associations. If the interaction term was significant for a given model, we presented the specific nutrient/food component–Klotho association stratified by gender. In models where the interaction term was not significant, the interaction term was omitted and the nutrient/food component–Klotho association for both genders was presented combined. Data were analyzed using SPSS Statistics for Mac (Version 24.0; IBM), with the significance level set at *p* < .05.

## 3 RESULTS

**FIGURE 1:**
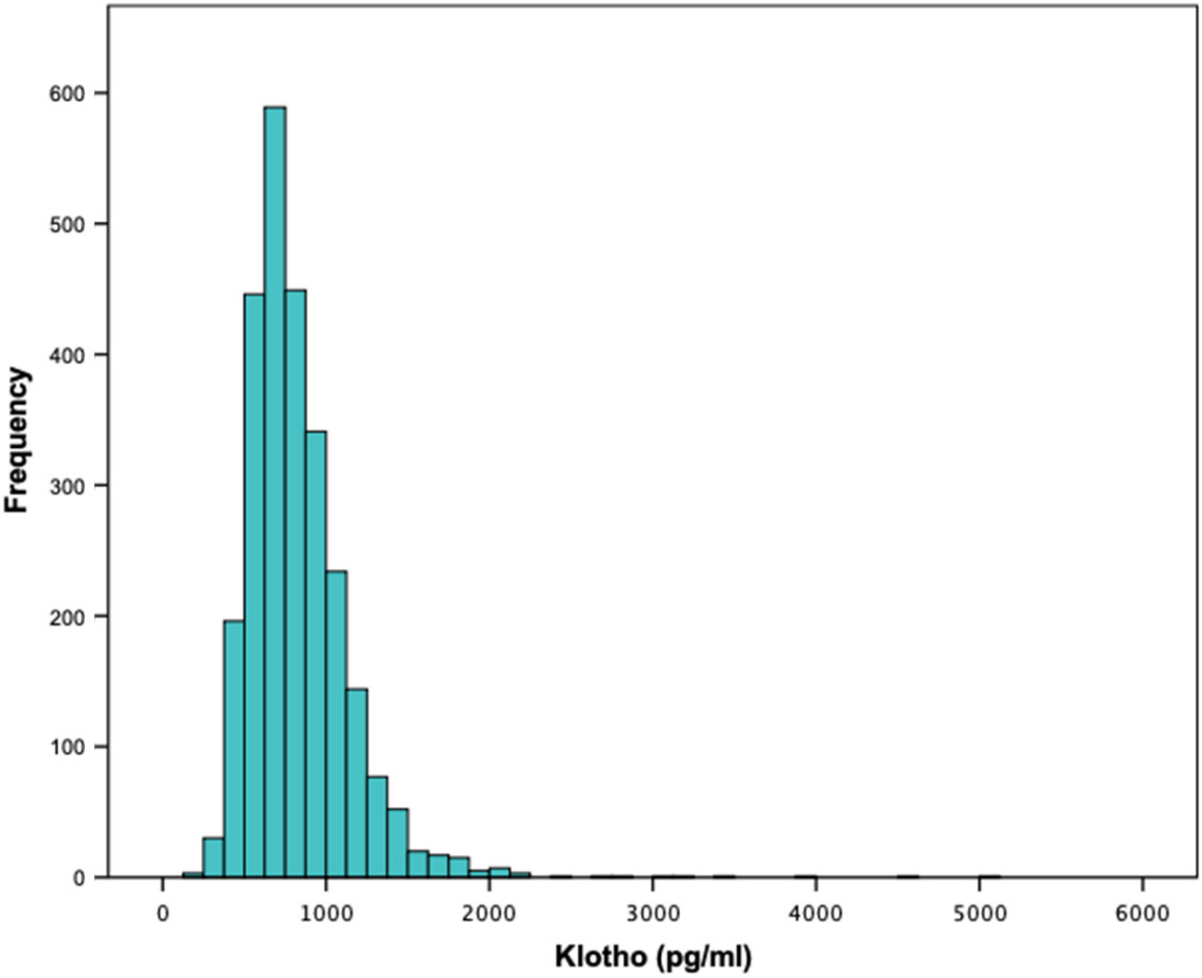
Histogram of serum Klotho levels in U.S. adults aged 40–79 years (*n* = 2637).

The final study sample consisted of 2637 participants (52% women) who provided dietary data and soluble Klotho levels (Flowchart, Supplementary material). The demographic and basic dietary characteristics of the study participants are shown in Table 1. The mean soluble serum Klotho level across all participants was 827.1 ± 324.9 pg/mL (95% confidence interval [CI] from 814.7 to 839.5) (Figure 1). Significantly lower serum Klotho levels were found in men than in women (797.4 ± 302.8 pg/mL vs. 854.4 ± 341.9 pg/mL; *p* < .001). Significant gender differences were found for energy‐adjusted intakes of several nutrients/food components (*p* < .05), including carbohydrates, alcohol, total sugars, dietary fibers, polyunsaturated fatty acids, alcohol, and several micronutrients and other food components (Table S1). For most nutrients/food components, women had a higher nutrient density than men.

**TABLE 1:** Demographic and basic dietary characteristics of the study sample.

	Total *n* = 2637
Age, mean (SD), years	58.0 (10.7)
Women, *n* (%)	1372 (52.0)
Body mass index, mean (SD), kg/m^2^	30.1 (6.8)
Race, %	
Mexican American	18.8
Other Hispanic	14.8
Non‐Hispanic White	33.8
Non‐Hispanic Black	18.8
Other Race, including multi‐racial	13.8
Educational level, %	
Less than 9th grade	14.7
9‐11th grade	11.6
High school graduate	22.1
Some college	27.6
College graduate or above	23.9
Annual family income, %	
$ 0 to $ 14,999	14.9
$ 15,000 to $ 44,999	32.5
$ 45,000 to $ 74,999	18.1
$ 75,000 and over	25.1
Energy intake, mean (SD), kcal/d	2027 (887)
Protein, mean (SD), g/d	79.1 (39.4)
Carbohydrate, mean (SD), g/d	239.0 (112.4)
Fat, total, mean (SD), g/d	79.5 (44.3)
Alcohol, mean (SD), g/d	9.3 (24.9)

**FIGURE 2:**
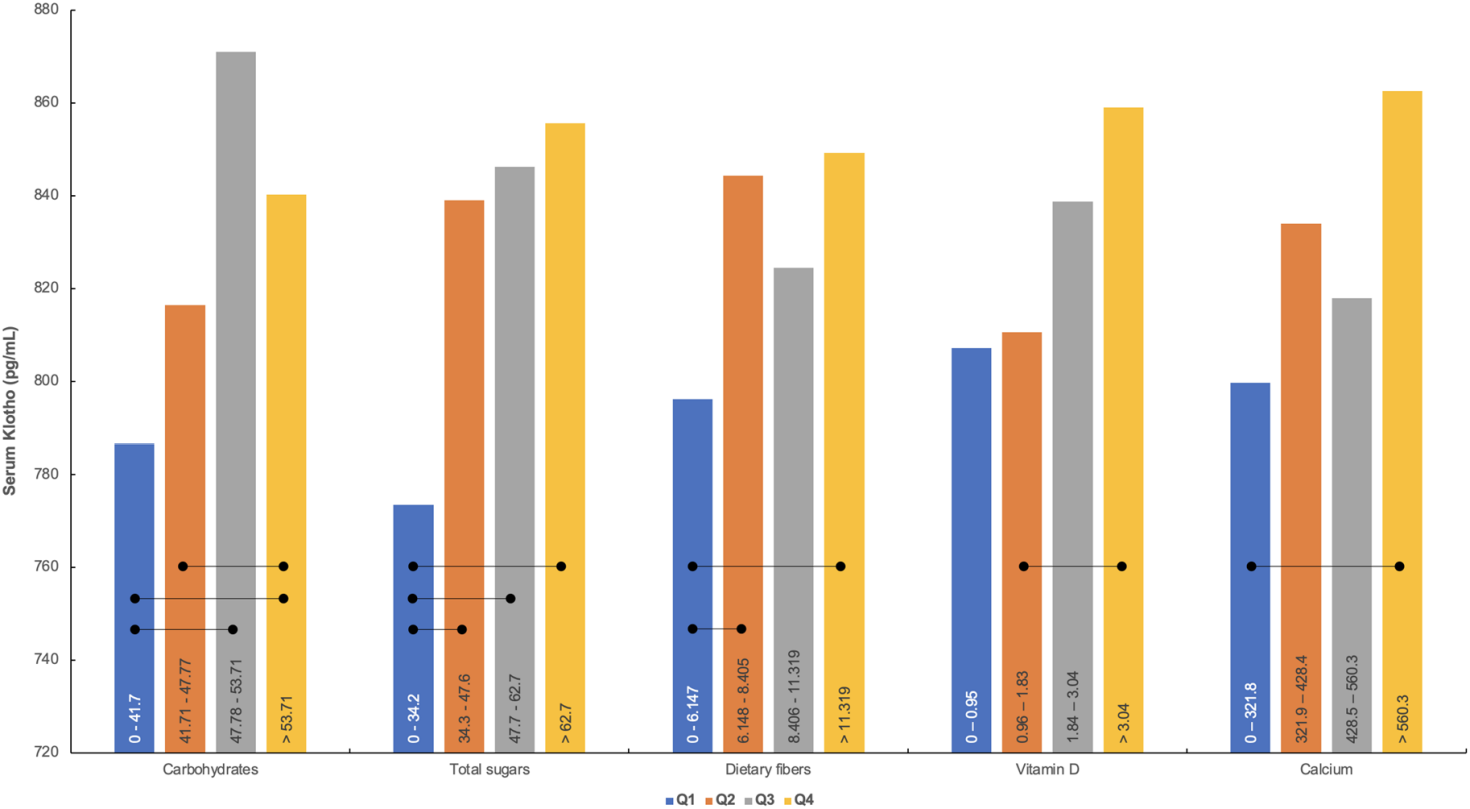
Mean serum Klotho concentrations across quartiles for intakes of carbohydrates (% of energy), total sugars (g/1000 kcal/day), dietary fibers (g/1000 kcal/day), vitamin D (mcg/1000 kcal/d), and calcium (mg/1000 kcal/d). The dietary intakes per quartile are depicted inside the bars for each nutrient. Error bars are removed for clarity. Rounded lines mark significant differences between quartiles at *p* < .05.

Mean serum Klotho levels varied across quartiles of dietary intake for several nutrients/food components, including carbohydrates, total sugars, dietary fibers, vitamin D, and calcium (Figure 2). A significant trend for higher Klotho levels with higher intake of a specific nutrient was found for carbohydrates (*p* < .001), total sugars (*p* < .001), dietary fibers (*p* < .001), vitamin D (*p* = .05), total folates (*p* = .015), and copper (*p* = .018) (Table S2). The participants who consumed over 1.39% of the total energy intake from alcohol per day (median intake) had significantly lower serum Klotho concentrations as compared to participants who consumed less alcohol (770.2 ± 313.2 pg/mL vs. 848.0 ± 331.8 pg/mL; *p* < .001). Serum Klotho levels across quartiles for other nutrients/food components are presented in Table S2.

**FIGURE 3:**
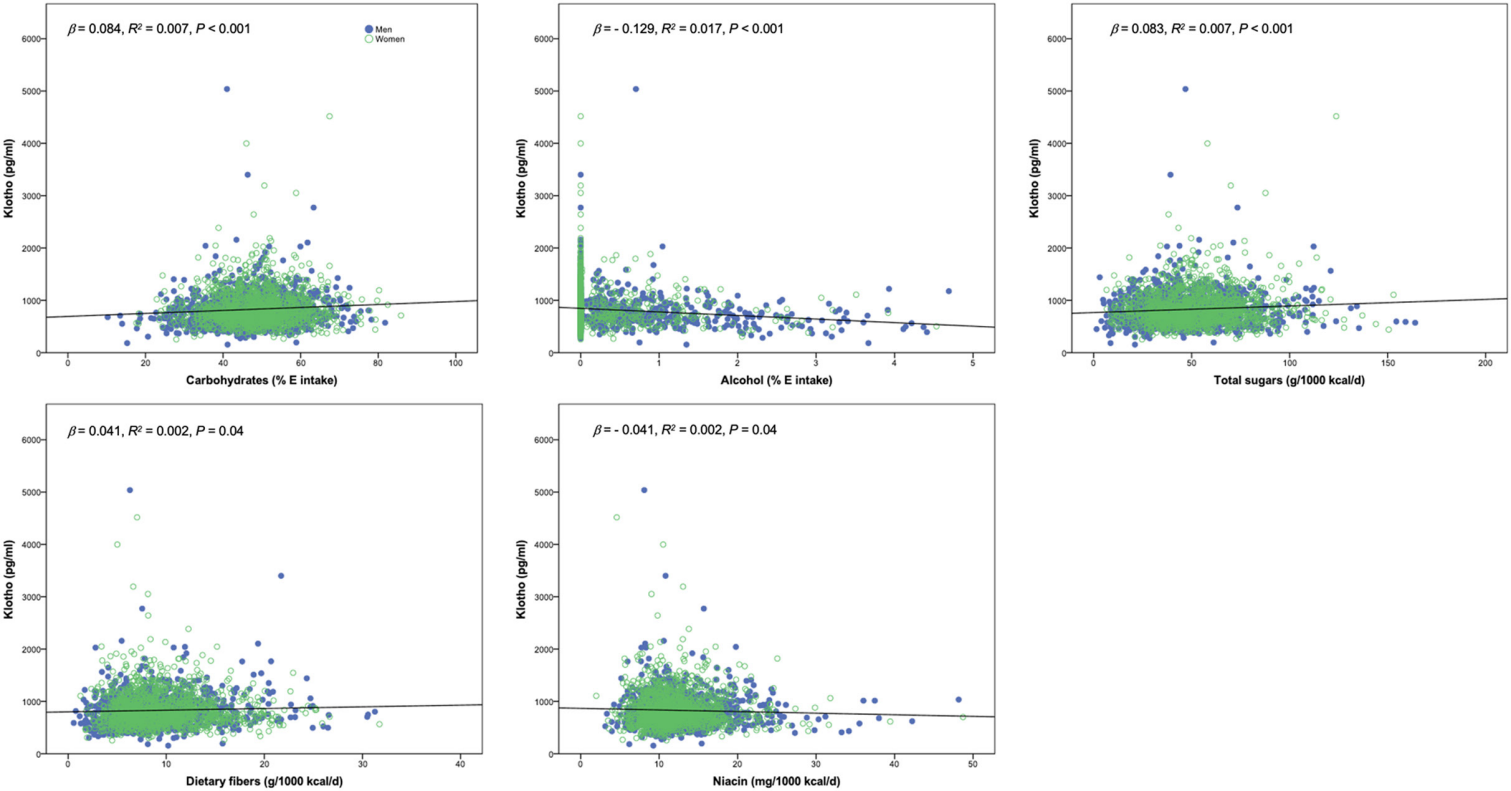
Simple (crude) regression models with significant association (*p* < .05) between specific nutrients/food components and serum Klotho levels in U.S. adults aged 40–79 years.

A bivariate analysis identified age (*r* = −.077; *p* < .001) and gender (*r* = .081; *p* < .001) as relevant covariates for a regression model, while other variables (e.g., race, educational level, family income, body mass index) did not provide a significant contribution to the model and were excluded for regression analyses (*p* > .05). The results of the regression analysis with a crude model showed a significant association among five nutrients/food components (carbohydrates, alcohol, total sugars, dietary fibers, and niacin) and soluble Klotho levels across the sample (Figure 3); the crude regression models for all nutrients and food components are presented in Table S3. We identified a significant interaction by gender on the nutrient–Klotho association for dietary fibers, phosphorus, and potassium. The specific regression coefficients stratified by gender are presented in Table 2, with significant associations found only in men (*p <* .05). In the other multivariable models, the interaction term was not significant, and the interaction was omitted from the models, with the nutrient/food component–Klotho association analyzed for both genders combined. After adjusting the models, we found that the nutrient/food component–Klotho association remained significant for carbohydrates, total sugars, and alcohol (*p* < .05) (Table S3).

**TABLE 2:** Adjusted regression model (adjusted for age) with significant interaction by gender on the energy‐adjusted nutrient/food component–Klotho association in men and women.

	B	*p*
Men		
Dietary fibers	0.128	<.001
Phosphorus	0.067	.020
Potassium	0.074	.011
Women		
Dietary fibers	−0.033	.238
Phosphorus	−0.016	.558
Potassium	−0.020	.471

## 4 DISCUSSION

The present study is, to our knowledge, the first cross‐sectional report that demonstrated significant associations between several individual nutrients or food components and serum Klotho levels at a populational level. We found that middle‐aged U.S. adults who report having a higher intake of carbohydrates, total sugars, and less alcohol have higher levels of circulating Klotho, compared to individuals with lower intakes of carbohydrates and total sugars, and more alcohol, after controlling for age and gender. Significantly lower Klotho levels were found in men than in women. Higher dietary intakes of dietary fibers, phosphorus, and potassium predicted higher serum Klotho only in men. Our findings suggest that exposure to several nutrients and food components may need to be considered when monitoring Klotho biodynamics in population studies.

Besides many other functions, the Klotho protein is often suggested as a main molecular suppressor of aging and perhaps a new therapeutic target for various antiaging interventions. Several cross‐sectional studies reported an inverse association between age and serum Klotho levels (Koyama et al., 2015; Martín‐Núñez et al., 2020). In addition, higher serum Klotho concentrations are reported to be associated with better cognition, psychological components of frailty, dependence, and less severe falls in the elderly (Sanz et al., 2021). Klotho may protect against age‐related conditions through multiple mechanisms, including optimal synaptic function promotion, stimulating the antioxidant defense system, reducing inflammation, and promoting autophagy (Hanson et al., 2021). Various dietary factors have been found to be associated with higher Klotho activity in preclinical, clinical, and small‐scope populational studies, including phosphate‐deficient diet (Hikone et al., 2017), diets enriched with keto‐analogs (Milovanova et al., 2017), high‐sucrose diet (Maekawa et al., 2017), calcium reduction (Wilkens et al., 2018), diet rich in nuts (Jurado‐Fasoli, Amaro‐Gahete, De‐la‐O, Martinez‐Tellez, et al., 2019), protein restriction (Zapata et al., 2019), low‐calorie high‐protein diets (Shafie et al., 2020), low‐salt diet (Hu et al., 2020), and vitamin D replacement (Dos Santos et al., 2021). The most comprehensive diet–Klotho study to date evaluated the relationship between dietary factors and soluble Klotho plasma levels in young (mean age 22.1 years) sedentary healthy adults (Jurado‐Fasoli et al., 2021). The authors reported an inverse association between Klotho levels and the dietary inflammatory index (a composite score of 28 nutrients; an individual's diet is considered more pro‐inflammatory when the score is higher) in 139 young men and women. The study also demonstrated no significant associations between total energy intake (also macronutrients) with Klotho plasma levels, except for a direct association found between alcohol intake and serum Klotho levels in women. Our study partially corroborates these previous findings, but we identified several additional energy‐adjusted nutrients and food components linked with serum Klotho levels and expand the research scope to middle‐aged adults.

In terms of macronutrients, we found a significant positive association between carbohydrates and total sugars and serum Klotho levels. Specifically, significantly higher Klotho concentrations in the fourth quartiles of dietary intake of carbohydrates (> 53.7% of energy) and total sugars (> 62.7 grams per 1000 kcal per day) were observed compared to the other quartiles. A recent trial observed a weak positive association between pro‐inflammatory diet (which accounts for carbohydrates consumed) and soluble Klotho plasma levels in a small sample of 40‐ to 65‐year‐old adults (Jurado‐Fasoli et al., 2020), yet the total intake of carbohydrates and sugars was not quantified in this study, and the association disappeared after controlling for body mass index. Our study demonstrated an association between energy‐adjusted carbohydrates/total sugars and soluble Klotho levels, even after adjusting for age and gender. A possible explanation for this interconnection could be related to the role of all factors in glucose turnover, with Klotho protein involved in the modulation of glucose homeostasis through signaling pathways involving insulin, insulin‐like growth factor (IGF‐1), and fibroblast growth factor 21 (FGF‐21) across various tissues (Gu et al., 2020; Wolf et al., 2008). Hypothetically, a higher intake of dietary carbohydrates might upregulate Klotho activity, either directly or through IGF‐1 and FGF‐21 axis modulation. This was preliminarily reported in an animal study (Maekawa et al., 2017), where a high‐sucrose diet was accompanied by increased Klotho mRNA expression in brown adipose tissue.

Higher alcohol intake was inversely associated with Klotho levels in our study, confirming the interrelationship reported in several previous studies (for a review, see Ref. (Martín‐González et al., 2019)), but not all (González‐Reimers et al., 2019). Being a pro‐inflammatory and pro‐oxidative agent, alcohol intake can contribute to loss of health across the lifespan. Its effects on Klotho downregulation could be mediated by a combination of oxidative stress, inflammation, and dehydration (Hu et al., 2013; Jurado‐Fasoli, Amaro‐Gahete, De‐la‐O, Gutiérrez, & Castillo, 2019; Tang et al., 2011), and perhaps affected by the amount of alcohol consumed (González‐Reimers et al., 2018).

The observed association between Klotho levels and dietary fiber, phosphorus, and potassium confined to male participants is of uncertain significance. Jurado‐Fasoli and co‐workers found no interaction of the diet–Klotho association by gender in a middle‐aged sample (Jurado‐Fasoli et al., 2021). Gender‐specific differences in diet–health outcomes are, however, not implausible (Vinke et al., 2020). Male participants in our sample had lower mean Klotho levels combined with lower nutrient density for dietary fiber, phosphorus, and potassium compared with women. Given the small effect sizes for phosphorus and potassium, these associations should be interpreted with caution. The positive association with dietary fiber was, however, stronger than the associations observed for carbohydrates and total sugars in the whole sample. We have no explanation for this but it may be that the association between dietary fiber intake and Klotho might plateau for dietary intake above a certain level in line with higher energy‐adjusted fiber intake among women. Potential gender differences in the observed diet–Klotho associations should be confirmed in future studies.

As opposed to the more robust findings observed for carbohydrates, including total sugars, and for alcohol in this study, the links between Klotho levels and the intake of dietary fibers, vitamin D, total folates, copper, and niacin must be interpreted with great caution. Our findings on dietary fiber remain unclear, as previously discussed. This is also the case for vitamin D, although Klotho's suggested role in calcium and phosphate homeostasis, through suppressing serum vitamin D in the form of 1,25(OH)(2)D has been known for more than a decade (Martin et al., 2012), also confirmed by the negative association between serum 1,25(OH)(2D) and Klotho levels recently observed in a sample of sedentary adults (De‐la‐O et al., 2021). In our study, there was a significant increase in serum Klotho levels across increasing quartiles of dietary intake of vitamin D, but we did only observe a borderline positive association between Klotho and vitamin D in both the crude and adjusted regression models. This potentially weak positive association is corroborated by the findings of a double‐blinded RCT in older adults (> 60 years) assessing the effect of vitamin D supplements on serum Klotho (Azimzadeh et al., 2020), but clearly needs to be confirmed in other studies. Moreover, when conducting multiple hypothesis tests at once, as done in our study, the risk of false‐positive results increases. Until future studies confirm that dietary fibers, vitamin D, total folates, copper, and niacin are associated with Klotho, we conjecture that there is no pertinent association.

Although we analyzed a relatively large and heterogeneous sample of middle‐aged US adults using an extensive list of independent variables (dietary exposures) and covariates, the present study is not without limitations. First, the cross‐sectional design of our study prohibits any assertion of causality. Second, a 24‐hour dietary recall used in this study to collect dietary information is based on self‐reports and prone to bias, and has low reliability at the individual level, due to large day‐to‐day variations in diet, and hence a two‐day intake does not capture the usual diet. This is especially a limitation for episodically consumed foods and beverages, like alcohol. On the contrary, the intake of carbohydrates, including total sugars, fluctuates less than alcohol on a day‐to‐day basis. Hence, the findings for carbohydrates and total sugars may be better grounded than the observations for alcohol. Furthermore, we analyzed a sample of middle‐aged adults which limits the interpretation of our findings to this population; additional studies are highly warranted to extrapolate our results to younger cohorts, elderly over 80 years, and clinical populations. Finally, well‐designed longitudinal observational studies including advanced biomarkers of Klotho activity and expression, in addition to detailed and rigorous dietary assessment, are necessary to advance our understanding of the possible effects of nutrients and food components on serum Klotho levels and health.

## 5 CONCLUSION

In conclusion, energy‐adjusted intake of carbohydrates, total sugars, and alcohol from a regular diet is associated with soluble Klotho serum levels in U.S. adults aged 40–79 years. Dietary exposure to individual nutritional components should be included in deciphering Klotho turnover in populational studies, with additional research needed to investigate the relationship between cause and effect in diet composition–Klotho interplay.

## AUTHOR CONTRIBUTIONS

The authors' responsibilities were as follows: Sergej M. Ostojic: Conceptualization; Data curation; Formal analysis; Investigation; Methodology; Project administration; Resources; Software; Supervision; Validation; Visualization; Writing‐original draft; Writing‐review and editing. Elisabet R. Hillesund: Conceptualization; Data curation; Formal analysis; Investigation; Methodology; Supervision; Validation; Writing‐review and editing. Nina C. Øverby: Investigation; Methodology; Project administration; Resources; Supervision; Validation; Writing‐review and editing. Frøydis N. Vik: Investigation; Methodology; Project administration; Resources; Supervision; Validation; Writing‐review and editing. Anine C. Medin: Conceptualization; Data curation; Formal analysis; Investigation; Methodology; Supervision; Validation; Visualization; Writing‐review and editing.

## FUNDING INFORMATION

None.

## CONFLICT OF INTEREST STATEMENT

Sergej M. Ostojic serves as a member of the Scientific Advisory Board on creatine in health and medicine (AlzChem LLC). Sergej M. Ostojic owns the patent “Supplements Based on Liquid Creatine” at European Patent Office (WO2019150323 A1), and the patent “Methods and Compositions for Improving a Response to a Metabolic Stress” at the United States Patent and Trademark Office (US 2015/0150933 A1). Sergej M. Ostojic has served as a speaker at Abbott Nutrition and has received research funding related to nutrition during the past 36 months from the World Health Organization, Serbian Ministry of Education, Science, and Technological Development, Provincial Secretariat for Higher Education and Scientific Research, Allied Beverages Adriatic, AlzChem GmbH, ThermoLife International, Hueston Hennigan, HRW Natural Health Products Inc, Aktivátor Kft, and CarnoMed. Sergej M. Ostojic does not own stocks and shares in any organization. Elisabet R. Hillesund, Nina C. Øverby, Frøydis N. Vik, and Anine C. Medin declare no known competing financial interests or personal relationships that could have appeared to influence the authorship of this paper.

## ETHICS STATEMENT

The ethical approval to conduct the current round of NHANES 2015–2016 was granted by the NCHS Research Ethics Review Board (Continuation of Protocol #2011–17).

## CONSENT TO PARTICIPATE STATEMENT

Informed consent was obtained from all respondents to participate in the study. The research was conducted ethically in accordance with the World Medical Association Declaration of Helsinki.

## Supporting information


Table S1.

## Data Availability

All data analyzed during this study are included in this article. Further enquiries can be directed to the corresponding author.
