# Supplementary material for: Individual nutrients and serum klotho levels in adults aged 40–79 years
Source: Food Sci Nutr. 2023 Mar 8;11(6):3279–86. doi: 10.1002/fsn3.3310 (PMC10261765; doi:10.1002/fsn3.3310)
Supplement: Supplementary file 1 — Table S1. [file FSN3-11-3279-s001.docx]

**Supplementary Table 1.** Mean differences in daily intakes of nutrients/food components adjusted for total energy intake stratified by gender. Values are mean ± SD. Asterisk (*) indicates a significant difference at *P* < 0.05

|  | Men  *n* = 1,199 | Women  *n* = 1,282 |
| --- | --- | --- |
| Energy (kcal/d) * | 2317 ± 959 | 1757 ± 715 |
|  |  |  |
| Protein (% of energy) | 16.2 ± 4.5 | 16.3 ± 4.3 |
| Carbohydrates (% of energy) * | 46.9 ± 10.0 | 48.7 ± 9.4 |
| Fat (% of energy) | 34.4 ± 8.2 | 34.9 ± 8.1 |
| Alcohol (% of energy) * | 3.6 ± 7.4 | 1.6 ± 4.7 |
|  |  |  |
| Water (g/1000 kcal/d) * | 1438.5 ± 762.3 | 1684.6 ± 989.9 |
|  |  |  |
| Total sugars (g/1000 kcal/d) * | 47.6 ± 22.2 | 51.8 ± 21.1 |
| Dietary fibers (g/1000 kcal/d) * | 8.6 ± 4.2 | 9.6 ± 4.2 |
|  |  |  |
| Total saturated fats (g/1000 kcal/d) | 12.3 ± 3.8 | 12.3 ± 3.8 |
| Total monounsaturated fats (g/1000 kcal/d) | 13.7 ± 3.9 | 13.7 ± 4.0 |
| Total polyunsaturated fats (g/1000 kcal/d) * | 8.8 ± 3.2 | 9.2 ± 3.2 |
| Cholesterol (mg/1000 kcal/d) | 153.1 ± 88.0 | 155.5 ± 87.8 |
|  |  |  |
| Retinol (mcg/1000 kcal/d) * | 189.5 ± 204.5 | 219.5 ± 385.4 |
| Carotenoids |  |  |
| Alpha-carotene (mcg/1000 kcal/d) | 222.6 ± 633.9 | 264.0 ± 767.2 |
| Beta-carotene (mcg/1000 kcal/d) * | 1067.3 ± 1635.7 | 1543.7 ± 2665.6 |
| Beta-cryptoxanthin (mcg/1000 kcal/d) * | 46.6 ± 98.2 | 68.0 ± 201.5 |
| Lycopene (mcg/1000 kcal/d) | 2454.8 ± 3637.8 | 2393.5 ± 3225.9 |
| Lutein + zeaxanthin (mcg/1000 kcal/d) * | 711.5 ± 1133.9 | 1129.0 ± 2434.3 |
| Vitamin E as alpha-tocopherol (mg/1000 kcal/d) * | 4.0 ± 2.1 | 4.4 ± 2.2 |
| Vitamin D (D2+D3) (mcg/1000 kcal/d) * | 2.3 ± 2.2 | 2.5 ± 2.5 |
| Vitamin K as phylloquinone (mcg/1000 kcal/d) * | 52.5 ± 56.7 | 74.7 ± 116.9 |
| Vitamin C (mg/1000 kcal/d) * | 38.7 ± 36.3 | 47.2 ± 40.5 |
| Thiamin (mg/1000 kcal/d) | 0.8 ± 0.3 | 0.8 ± 0.3 |
| Riboflavin (mg/1000 kcal/d) | 1.0 ± 0.4 | 1.0 ± 0.4 |
| Niacin (mg/1000 kcal/d) * | 12.7 ± 4.6 | 12.1 ± 4.2 |
| Vitamin B6 (mg/1000 kcal/d) | 1.0 ± 0.5 | 1.0 ± 0.4 |
| Total folate (mcg/1000 kcal/d) * | 191.2 ± 84.8 | 199.9 ± 81.5 |
| Total choline (mcg/1000 kcal/d) | 172.4 ± 62.9 | 172.7 ± 65.1 |
| Vitamin B12 (mcg/1000 kcal/d) | 2.3 ± 1.9 | 2.5 ± 4.1 |
|  |  |  |
| Calcium (mg/1000 kcal/d) * | 433.5 ± 187.7 | 479.7 ± 196.7 |
| Iron (mg/1000 kcal/d) | 7.0 ± 2.9 | 7.1 ± 2.8 |
| Magnesium (mg/1000 kcal/d) * | 148.6 ± 48.9 | 161.5 ± 52.9 |
| Phosphorus (mg/1000 kcal/d) * | 664.6 ± 151.8 | 679.4 ± 155.0 |
| Potassium (mg/1000 kcal/d) * | 1311.7 ± 383.0 | 1396.7 ± 420.6 |
| Sodium (mg/1000 kcal/d) | 1705.8 ± 475.3 | 1723.1 ± 529.8 |
| Zinc (mg/1000 kcal/d) | 5.5 ± 2.7 | 5.4 ± 2.3 |
| Copper (mg/1000 kcal/d) | 0.6 ± 0.3 | 0.7 ± 0.7 |
| Selenium (mcg/1000 kcal/d) | 57.6 ± 18.3 | 57.2 ± 17.7 |
|  |  |  |
| Caffeine (mg/1000 kcal/d) | 85.5 ± 115.1 | 83.5 ± 96.8 |
| Theobromine (mg/1000 kcal/d) * | 11.8 ± 24.0 | 15.1 ± 26.1 |

**Supplementary Table 2.** Mean serum Klotho levels across quartiles for energy and various nutrients/food components (values are mean ± SD). Asterisk (*) indicates a significant difference between quartiles at *P* < 0.05, with superscript letters indicated post-hoc differences between Q1 *vs.* Q2 (^a^), Q1 *vs.* Q3 (^b^), Q1 *vs.* Q4 (c), and Q2 *vs.* Q3 9 (^d^), Q2 vs Q4 (^e^). Cross (†) indicates significant trend at *P* < 0.05.

|  | **Serum Klotho levels (pg/mL)** | | | |
| --- | --- | --- | --- | --- |
|  | Q1 | Q2 | Q3 | Q4 |
| Energy, kcal/day  Q1 (0 – 1424)  Q2 (1425 – 1892)  Q3 (1893 – 2373)  Q4 (> 2373) | 839.3 ± 355.8 | 836.9 ± 334.3 | 816.0 ± 279.9 | 822.0 ± 340.8 |
|  |  |  |  |  |
| Protein, % energy intake  Q1 (0 – 13.3)  Q2 (13.4 – 15.6)  Q3 (15.7 – 18.5)  Q4 (> 18.5) | 832.0 ± 353.0 | 832.1 ± 363.7 | 824.0 ± 286.7 | 826.1 ± 307.0 |
| Carbohydrates, % energy intake ^* b c d †^  Q1 (0 – 41.7)  Q2 (41.8– 47.8)  Q3 (47.9 – 53.7)  Q4 (> 0.5371) | 786.6 ± 326.5 | 816.4 ± 316.5 | 871.0 ± 335.3 | 840.2 ± 332.1 |
| Fat, % energy intake  Q1 (0 – 29.4)  Q2 (29.5 – 34.9)  Q3 (34.9 – 39.9)  Q4 (> 39.9) | 812.9 ± 322.7 | 841.5 ± 318.1 | 845.5 ± 377.8 | 814.2 ± 290.4 |
| Alcohol, % energy intake ^* †^  M1 (0 – 1.39)  M2 (> 1.39) |  | 848.0 ± 331.8 | 770.2 ± 313.2 |  |
|  |  |  |  |  |
| Water, g/1000 kcal/d  Q1 (0 – 1015)  Q2 (1016 – 1349)  Q3 (1350 – 1877)  Q4 (> 1877) | 835.5 ± 338.0 | 838.0 ± 324.9 | 817.2 ± 309.4 | 823.4 ± 342.8 |
|  |  |  |  |  |
| Total sugars, g/1000 kcal/d ^* a b c †^  Q1 (0 – 34.2)  Q2 (34.3 – 47.6)  Q3 (47.7 – 62.7)  Q4 (> 0.627) | 773.5 ± 253.6 | 839.0 ± 363.3 | 846.3 ± 318.9 | 855.6 ± 362.2 |
| Dietary fibers, g/1000 kcal/d ^* a c †^  Q1 (0 – 6.2)  Q2 (6.2 – 8.4)  Q3 (8.5 – 11.3)  Q4 (> 11.319) | 796.1 ± 311.9 | 844.4 ± 400.8 | 824.5 ± 277.5 | 849.2 ± 310.9 |
|  |  |  |  |  |
| Total saturated fats, g/1000 kcal/d  Q1 (0 – 9.8)  Q2 (9.9 – 12.1)  Q3 (12.2 – 14.7)  Q4 (> 14.7) | 819.7 ± 334.9 | 838.7 ± 316.0 | 834.5 ± 319.6 | 821.2 ± 345.3 |
| Total monounsaturated fats, g/1000 kcal/d  Q1 (0 – 11.1)  Q2 (11.2 – 13.4)  Q3 (13.5 – 16.0)  Q4 (> 16.0) | 816.1 ± 333.3 | 844.2 ± 311.0 | 826.7 ± 362.4 | 826.7 ± 306.7 |
| Total polyunsaturated fats, g/1000 kcal/d  Q1 (0 – 6.8)  Q2 (6.9 – 8.6)  Q3 (8.7 – 10.7)  Q4 (> 10.7) | 827.3 ± 323.1 | 835.2 ± 347.8 | 827.7 ± 330.6 | 823.9 ± 314.3 |
| Cholesterol, mg/1000 kcal/d  Q1 (0 – 92.8)  Q2 (92.9 – 134.5)  Q3 (134.6 – 196.8)  Q4 (> 196.8) | 836.5 ± 358.5 | 820.8 ± 299.5 | 824.1 ± 337.9 | 832.8 ± 317.5 |
| Retinol, mcg/1000 kcal/d  Q1 (0 – 104.8)  Q2 (104.9 – 162.3)  Q3 (162.4 – 240.7)  Q4 (> 240.7) | 826.9 ± 333.4 | 814.7 ± 352.8 | 820.6 ± 276.7 | 851.9 ± 346.9 |
| Alpha-carotene, mcg/1000 kcal/d  Q1 (0 – 129.7)  Q2 (129.8 – 481.0)  Q3 (481.1 – 2487.4)  Q4 (> 2487.4) | 829.5 ± 357.7 | 819.0 ± 282.5 | 848.0 ± 381.9 | 817.6 ± 281.4 |
| Beta-carotene, mcg/1000 kcal/d  Q1 (0 – 239.7)  Q2 (239.8 – 593.2)  Q3 (593.3 – 1503.0)  Q4 (> 1503.0) | 848.6 ± 416.8 | 822.2 ± 297.0 | 820.3 ± 296.6 | 823.0 ± 287.8 |
| Beta-cryptoxanthin, mcg/1000 kcal/d  Q1 (0 – 8.8)  Q2 (8.9 – 24.0)  Q3 (24.1 – 57.7)  Q4 (> 57.7) | 829.7 ± 327.9 | 821.5 ± 312.7 | 831.9 ± 351.2 | 831.0 ± 323.5 |
| Lycopene, mcg/1000 kcal/d  Q1 (0 – 352.5)  Q2 (325.6 – 1239.6)  Q3 (1239.7 – 3146.2)  Q4 (> 3146.2) | 821.5 ± 340.7 | 828.6 ± 318.2 | 834.2 ± 353.9 | 829.9 ± 301.1 |
| Lutein plus zeaxanthin, mcg/1000 kcal/d  Q1 (0 – 258.3)  Q2 (258.4 – 441.2)  Q3 (441.2 – 844.6)  Q4 (> 844.6) | 823.2 ± 372.6 | 823.1 ± 334.7 | 835.2 ± 299.8 | 832.7 ± 304.0 |
| Vitamin E as alpha-tocopherol, mg/1000 kcal/d  Q1 (0 – 3.0)  Q2 (3.1 – 3.8)  Q3 (3.9 – 4.9)  Q4 (> 4.9) | 829.2 ± 357.9 | 818.2 ± 281.9 | 822.8 ± 317.3 | 843.7 ± 346.8 |
| Vitamin D (D2+D3), mcg/1000 kcal/d * ^e^ ^†^  Q1 (0 – 0.95)  Q2 (0.96 – 1.83)  Q3 (1.84 – 3.04)  Q4 (> 3.04) | 807.1 ± 294.2 | 810.5 ± 295.8 | 838.7 ± 356.4 | 859.0 ± 361.3 |
| Vitamin K as phylloquinone, mcg/1000 kcal/d  Q1 (0 – 25.8)  Q2 (25.9 – 39.8)  Q3 (39.9 – 66.9)  Q4 (> 66.9) | 826.2 ± 357.8 | 841.4 ± 338.8 | 818.9 ± 302.3 | 827.6 ± 314.4 |
| Vitamin C, mg/1000 kcal/d  Q1 (0 – 15.9)  Q2 (16.0 – 32.5)  Q3 (32.6 – 58.3)  Q4 (> 58.3) | 810.1 ± 305.3 | 835.2 ± 404.5 | 827.9 ± 295.3 | 841.0 ± 297.9 |
| Thiamin, mg/1000 kcal/d  Q1 (0 – 0.61  Q2 (0.62 – 0.75)  Q3 (0.76 – 0.91)  Q4 (> 0.91) | 814.4 ± 332.5 | 838.8 ± 350.5 | 845.8 ± 332.5 | 815.2 ± 297.7 |
| Riboflavin, mg/1000 kcal/d  Q1 (0 – 0.77)  Q2 (0.78 – 0.95)  Q3 (0.96 – 1.19)  Q4 (> 1.19) | 809.5 ± 302.7 | 821.9 ± 307.8 | 845.3 ± 341.5 | 837.6 ± 360.0 |
| Niacin, mg/1000 kcal/d  Q1 (0 – 9.6)  Q2 (9.7 – 11.6)  Q3 (11.7 – 14.3)  Q4 (> 14.3) | 847.3 ± 388.8 | 830.0 ± 333.8 | 830.4 ± 292.5 | 806.4 ± 290.4 |
| Vitamin B6, mg/1000 kcal/d  Q1 (0 – 0.71)  Q2 (0.72 – 0.91)  Q3 (0.92 – 1.17)  Q4 (> 1.17) | 844.5 ± 392.3 | 821.6 ± 308.9 | 812.4 ± 259.1 | 835.7 ± 341.0 |
| Total folate, mcg/1000 kcal/d ^†^  Q1 (0 – 140.0)  Q2 (140.1 – 178.7)  Q3 (178.8 – 232.5)  Q4 (> 232.5) | 803.1 ± 318.9 | 830.1 ± 363.4 | 851.8 ± 310.5 | 829.2 ± 319.4 |
| Total choline, mcg/1000 kcal/d  Q1 (0 – 126.7)  Q2 (126.8 – 162.1)  Q3 (162.2 – 204.5)  Q4 (> 204.5) | 838.7 ± 351.2 | 818.4 ± 317.4 | 824.8 ± 329.5 | 832.3 ± 317.0 |
| Vitamin B12, mcg/1000 kcal/d  Q1 (0 – 1.27)  Q2 (1.28 – 1.87)  Q3 (1.88 – 2.74)  Q4 (> 2.74) | 812.1 ± 296.0 | 834.8 ± 333.9 | 835.2 ± 336.7 | 832.5 ± 329.0 |
|  |  |  |  |  |
| Calcium,mg/1000 kcal/d ^* c^  Q1 (0 – 321.8)  Q2 (321.9 – 428.4)  Q3 (428.5 – 560.3)  Q4 (> 560.3) | 799.7 ± 279.9 | 834.0 ± 311.6 | 817.9 ± 288.4 | 862.6 ± 415.6 |
| Iron, mg/1000 kcal/d  Q1 (0 – 5.3)  Q2 (5.4 – 6.5)  Q3 (6.6 – 8.0)  Q4 (> 8.0) | 813.7 ± 325.7 | 838.1 ± 355.8 | 825.8 ± 292.5 | 836.7 ± 338.8 |
| Magnesium, mg/1000 kcal/d  Q1 (0 – 119.1)  Q2 (119.2 – 147.3)  Q3 (147.4 – 181.9)  Q4 (> 181.9) | 818.0 ± 349.5 | 818.4 ± 316.1 | 833.3 ± 336.1 | 844.4 ± 312.8 |
| Phosphorus, mg/1000 kcal/d  Q1 (0 – 567.5)  Q2 (567.6 – 659.5)  Q3 (659.6 – 759.4)  Q4 (> 759.4) | 818.5 ± 348.5 | 835.0 ± 299.0 | 820.7 ± 327.9 | 840.0 ± 338.6 |
| Potassium, mg/1000 kcal/d  Q1 (0 – 1076)  Q2 (1077 - 1304)  Q3 (1305 – 1566)  Q4 (> 1566) | 823.7 ± 379.8 | 821.6 ± 303.1 | 833.9 ± 321.4 | 834.9 ± 306.3 |
| Sodium, mg/1000 kcal/d  Q1 (0 – 1407.4)  Q2 (1407.5 – 1652.8)  Q3 (1652.9 – 1940.3)  Q4 (> 1940.3) | 840.7 ± 359.7 | 819.0 ± 325.1 | 844.8 ± 343.7 | 809.7 ± 281.6 |
| Zinc, mg/1000 kcal/d  Q1 (0 – 4.2)  Q2 (4.3 – 5.0)  Q3 (5.1 – 6.2)  Q4 (> 6.2) | 835.4 ± 359.2 | 824.5 ± 294.5 | 825.1 ± 326.5 | 829.2 ± 333.0 |
| Copper, mg/1000 kcal/d ^†^  Q1 (0 – 0.47)  Q2 (0.48 – 0.58)  Q3 (0.59 – 0.72)  Q4 (> 0.72) | 814.4 ± 356.9 | 811.2 ± 297.0 | 835.2 ± 339.0 | 853.5 ± 318.8 |
| Selenium, mcg/1000 kcal/d  Q1 (0 – 45.3)  Q2 (45.4 – 55.0)  Q3 (55.1 – 66.5)  Q4 (> 66.5) | 839.3 ± 371.3 | 818.7 ± 352.9 | 840.2 ± 304.3 | 815.9 ± 278.9 |
|  |  |  |  |  |
| Caffeine, mg/1000 kcal/d  Q1 (0 – 18.2)  Q2 (18.3 – 57.7)  Q3 (57.8 – 114.7)  Q4 (> 114.7) | 834.1 ± 343.3 | 841.5 ± 370.0 | 832.5 ± 317.2 | 806.1 ± 278.0 |
| Theobromine, mg/1000 kcal/d  M1 (0 – 24.4)  M2 (> 24.4) |  | 837.8 ± 325.6 | 819.3 ± 332.2 |  |

### Supplementary Table 3. Simple (crude) and adjusted regression models between energy-adjusted nutrients/food components and serum Klotho levels with standardized β coefficient. Red letters indicate nutrients/food components where potential interactions by gender on the dietary constituent-Klotho associations were identified in an adjusted model, please refer to Table 2 for gender-specific adjusted regression model outcomes.

|  | Crude model | | Adjusted model | |
| --- | --- | --- | --- | --- |
|  | *b* | *P* | *b* | *P* |
| Protein | 0.003 | 0.883 | 0.003 | 0.894 |
| Carbohydrates | 0.084 | < 0.001 | 0.079 | < 0.001 |
| Fat | 0.000 | 0.994 | 0.002 | 0.927 |
| Alcohol | - 0.129 | < 0.001 | - 0.127 | < 0.001 |
|  |  |  |  |  |
| Water | - 0.008 | 0.685 | - 0.020 | 0.313 |
|  |  |  |  |  |
| Total sugars | 0.083 | < 0.001 | 0.075 | < 0.001 |
| Dietary fibers | 0.041 | 0.041 | 0.041 | 0.043 |
|  |  |  |  |  |
| Total saturated fats | - 0.004 | 0.839 | - 0.002 | 0.935 |
| Total monounsaturated fats | 0.003 | 0.880 | 0.008 | 0.701 |
| Total polyunsaturated fats | 0.003 | 0.871 | 0.000 | 0.981 |
| Cholesterol | -0.007 | 0.718 | -0.006 | 0.746 |
|  |  |  |  |  |
| Vitamin A as retinol activity equivalents | 0.009 | 0.661 | 0.008 | 0.681 |
| Retinol | -0.006 | 0.770 | -0.005 | 0.805 |
|  |  |  |  |  |
| Alpha-carotene | 0.021 | 0.294 | 0.021 | 0.303 |
| Beta-carotene | 0.024 | 0.227 | 0.022 | 0.278 |
| Beta-cryptoxanthin | 0.000 | 0.996 | - 0.004 | 0.861 |
| Lycopene | -0.030 | 0.137 | - 0.030 | 0.136 |
| Lutein + zeaxanthin | -0.009 | 0.657 | - 0.013 | 0.513 |
| Vitamin E as alpha-tocopherol | 0.024 | 0.232 | 0.015 | 0.445 |
| Vitamin D (D2+D3) | 0.037 | 0.062 | 0.038 | 0.057 |
| Vitamin K as phylloquinone | -0.009 | 0.637 | - 0.015 | 0.471 |
| Vitamin C | 0.028 | 0.170 | 0.022 | 0.265 |
| Thiamin (mg/1000 kcal/d) | -0.008 | 0.683 | - 0.003 | 0.863 |
| Riboflavin | 0.008 | 0.689 | 0.011 | 0.593 |
| Niacin | -0.041 | 0.043 | - 0.037 | 0.063 |
| Vitamin B6 | -0.030 | 0.131 | - 0.029 | 0.149 |
| Total folate | 0.004 | 0.841 | 0.002 | 0.917 |
| Total choline | -0.007 | 0.738 | - 0.002 | 0.909 |
| Vitamin B12 | -0.001 | 0.962 | - 0.001 | 0.950 |
|  |  |  |  |  |
| Calcium | 0.035 | 0.078 | 0.028 | 0.166 |
| Iron | -0.004 | 0.839 | 0.000 | 0.997 |
| Magnesium | 0.026 | 0.201 | 0.021 | 0.298 |
| Phosphorus | 0.022 | 0.266 | 0.021 | 0.294 |
| Potassium | 0.017 | 0.406 | 0.020 | 0.329 |
| Sodium | -0.032 | 0.112 | -0.032 | 0.110 |
| Zinc | -0.028 | 0.169 | -0.023 | 0.241 |
| Copper | 0.008 | 0.681 | 0.005 | 0.803 |
| Selenium | -0.016 | 0.431 | - 0.016 | 0.413 |
|  |  |  |  |  |
| Caffeine | -0.020 | 0.313 | -0.017 | 0.409 |
| Theobromine | 0.016 | 0.424 | 0.010 | 0.614 |
